# Discrepancies in Subjective Sleep Quality Between Home and Hospital Settings: Insights of Hypnotic Agents Use with Post-Polysomnography Questionnaire

**DOI:** 10.3390/diagnostics15243153

**Published:** 2025-12-11

**Authors:** Jing-Jie Wang, Ming-Feng Wu

**Affiliations:** 1 School of Medicine, College of Medicine, National Yang Ming Chiao Tung University, Taipei 112304, Taiwan; drshepherd2011@gmail.com; 2 Department of Otolaryngology, Taichung Veterans General Hospital, Taichung 407219, Taiwan; 3 Department of Chest Medicine, Taichung Veterans General Hospital, Taichung 407219, Taiwan; 4 Department of Medical Laboratory Science and Biotechnology, Central Taiwan University of Science and Technology, Taichung 406053, Taiwan

**Keywords:** polysomnography, sleep-disordered breathing, first-night effect, perceived sleep quality, home sleep apnea testing

## Abstract

**Background/Objectives:** Polysomnography (PSG) is the gold standard for diagnosing sleep-disordered breathing (SDB). However, in a hospital testing setting, it may produce the first-night effect, viz., prolonged sleep latency, lower sleep efficiency, and uncertain apnea–hypopnea index (AHI). Here, we aim to determine the influences of hypnotic agents and gender. **Methods:** In this retrospective study, we reviewed the post-PSG questionnaires and electronic medical records of patients aged ≥20 years receiving overnight PSG for the diagnosis of SDB at Taichung Veterans General Hospital in a period between April 2024 and March 2025. **Results:** We studied a total of 1053 patients, aged 47.0 ± 14.7 years old. Compared to sleeping at home, 42.2% of patients reported worse perceived sleep quality (PSQ) with hypnotic agents, and 53.0%, without, before PSG testing in the hospital. For those without taking hypnotic agents, men had an odds ratio (OR) of 1.570 (95% CI: 1.127–2.189) for worsening PSQ compared to women (*p* = 0.008). Also, per increasing 1 Epworth Sleepiness Scale (ESS) score, the risk of worsening PSQ was reduced by OR of 0.963 (95% CI: 0.933–0.994) (*p* = 0.021). Once male patients had ESS < 10, as many as 57.3% of them reported the worse PSQ. **Conclusions:** Our study suggests a potentially more patient-centric approach to diagnosing sleep-disordered breathing. In some male patients with ESS scores < 10, short-acting hypnotics might be considered during in-lab PSG to improve subjective comfort, thereby potentially enhancing study reliability. Meanwhile, home sleep apnea testing can serve as a practical initial tool for selected patients—offering convenience, mitigating the first-night effect, and potentially reducing long wait times. However, its use in individuals with comorbid insomnia requires careful clinical judgment to avoid false-negative results, often making in-lab assessment the preferred option in such cases.

## 1. Introduction

Polysomnography (PSG) is the most widely used as the diagnosing tool for sleep-disordered breathing (SDB), including obstructive sleep apnea (OSA) [1,2]. PSG comprehensively monitors various physiological parameters during sleep to determine relevant indices, like the apnea–hypopnea index (AHI) [3]. Despite its diagnostic value, PSG testing in a hospital environment often faces significant challenges. Since patients often report discomfort and difficulty sleeping in unfamiliar environments, the resulting sleep status determined is likely not identical to that determined in their home setting. These discrepancies could cause patient distrust in the hospital results and their reluctance to accept the necessary treatment [4,5].

The unstable PSG results are primarily related to the first-night effect (FNE) and inherent night-to-night variability [6,7]. The FNE, a well-documented phenomenon since 1964, refers to alterations in sleep architecture during the initial night of PSG, consisting typically a number of features, like prolonged sleep latency (SL), increased wake time after sleep onset (WASO), reduced total sleep time (TST), lower sleep efficiency (SE), less rapid eye movement (REM) sleep, and longer REM latency [6,8]. These changes are largely attributed to the discomfort from attached electrodes, restricted movement due to connecting cables, and psychological anxiety associated with being monitored in an unfamiliar environment [6,7]. The effects of FNE vary, being less pronounced in young adults (aged 20 to 29 years) but more significant in children, adolescents, middle-aged, and older adults [6]. Besides FNE, the night-to-night variability in sleep parameters, especially AHI, further complicates an accurate diagnosis. Studies reported that a single night PSG is insufficient to precisely assess conditions like OSA, with significant AHI fluctuations observed across consecutive nights [7,9].

The discrepancy between subjective and objective sleep assessments is a common issue for patients in a sleep laboratory [10,11]. Patients often report poor sleep quality during the laboratory study, and they believe the results are not credible and would not accept later treatment options. Subjective sleep quality refers to an individual’s subjective perception of their sleep quality, while objective sleep quality is measured by physiological means like PSG, including metrics such as SL, TST, SE, and sleep stage proportions [5,12].

During PSG, various factors change objective and subjective sleep quality. Studies showed that older age, male gender, OSA severity, and higher body mass index (BMI) are associated with shorter TST and lower SE [2,4]. In OSA patients, chronic heart failure and higher Epworth Sleepiness Scale (ESS) scores are linked to poorer subjective sleep quality [2]. Environmental factors such as high temperature, humidity, CO_2_, and PM2.5 can also significantly impair sleep quality [13]. In specific OSA patient subgroups with low arousal thresholds, some sedatives like eszopiclone increase arousal thresholds and improve sleep duration and AHI. Research has cautioned against routinely prescribing sleep medications to improve PSG results [14].

Therefore, a deeper understanding of the factors contributing to unstable PSG results and how these factors influence patient perception is critical. In this study, we aimed to cross-analyze differences in sleep latency, sleep efficiency, and total sleep time between the hospital and home settings, and to identify personal characteristics susceptible to environmental influences. The analysis was based on subjective post-polysomnography questionnaires for enhancing the stability and persuasiveness of diagnostic reports and to foster better patient trust and treatment adherence by allowing for pre-emptive health education and potentially personalized interventions for patients undergoing PSG.

## 2. Materials and Methods

### 2.1. Study Design and Population

In this retrospective study, we reviewed electronic medical records at the Taichung Veterans General Hospital between April 2024 and March 2025. Adults aged ≥20 years receiving overnight PSG study for the evaluation of SDB were eligible for study inclusion. Patients referred during hospitalization were excluded, as were those with incomplete physiological recordings or missing post-PSG questionnaires. Our study protocol was approved by the Institutional Review Board and Ethics Committee of Taichung Veterans General Hospital (approval number: CE25326B).

### 2.2. Data Collection

Baseline clinically relevant data were collected from electronic medical records for each patient. Subjective sleep perception was evaluated using a post-PSG questionnaire that compared the in-hospital sleep experience to usual sleep at home. Items included the following: (1) length of time to fall asleep (LTFA), categorized as either (a) same or shorter, or (b) longer; (2) perceived sleep quality (PSQ), categorized as either (a) same or better, or (b) worse; and (3) perceived sleep duration time (PSDT), categorized as either (a) same or longer, or (b) shorter. The use of hypnotic medications prior to PSG was also recorded. Objective sleep parameters were obtained from PSG data which included sleep duration time (SDT), sleep latency (SL), sleep efficiency (SE), and the percentage of slow-wave sleep (N3). We also collected apnea–hypopnea index (AHI) data to enable comparison of sleep-breathing disorder severity among subjects. Additional participant characteristics—age, gender, body mass index (BMI), and cardiovascular comorbidities—were also recorded.

### 2.3. Parameter Definitions

Total recording time (TRT) was defined as the interval from “light-off” to “light-on” during PSG. Sleep latency was defined as the time from “light-off” to sleep onset. Sleep efficiency was calculated as the ratio of TST to TRT. The proportion of deep sleep was expressed as the percentage of stage N3 duration relative to TST. REM sleep was expressed as the percentage of REM sleep duration relative to TST. The arousal index was defined as the number of EEG arousals per hour of sleep.

### 2.4. Statistical Analyses

Descriptive statistics were reported as mean and standard deviation for continuous variables, and as frequency with percentage for categorical variables. Inter-group differences for continuous variables were assessed using independent-samples *t*-test, while the chi-square test was applied to examine associations between questionnaire responses and participant characteristics. The logistic regression analysis was performed to evaluate the impact of significant parameters. A two-sided *p* value < 0.05 was considered statistically significant.

## 3. Results

**Figure 1:**
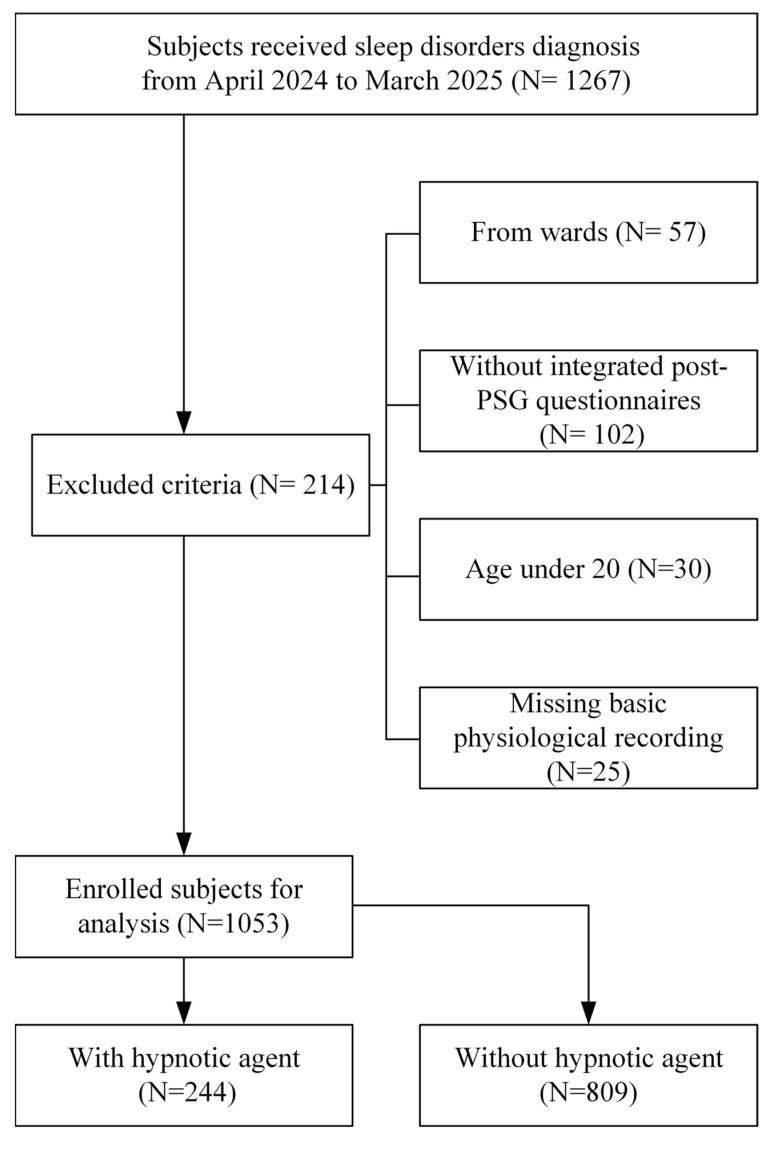
Patient enrollment flow chart.

A total of 1053 participants were included in the final analysis (Figure 1). The mean age was 47.0 ± 14.7 years (Table 1). The mean BMI was 25.6 ± 4.6 kg/m^2^, and the mean ESS score was 9.5 ± 4.4.

Based on the post-PSG questionnaires, 244 participants received hypnotic medications before the PSG examination. Comparisons between groups revealed significant differences in SDT, SL, and SE. Specifically, the hypnotic-agent group showed shorter SDT, longer SL, and lower SE compared with the non–hypnotic-agent group (all with *p* < 0.05).

Compared with sleep at home, 42.2% of participants receiving hypnotic agents before PSG indicated worse PSQ at the hospital, while the remainder reported the same or better PSQ. Based on post-questionnaires, the types of hypnotic agents administered before PSG diagnosis were investigated, demonstrating no significant difference in PSQ scores across Benzodiazepines (BZD), Z-drugs, and combined-hypnotic therapy (Supplementary Table S1 and Figure S1). In contrast, among participants without hypnotic agents, 53.0% reported worse PSQ during in-hospital PSG. This difference was statistically significant (*p* = 0.004). However, no significant differences were observed between groups in LTFA or PSDT.

For participants who did not take hypnotic agents before PSG testing, 278 males (73.2%) and 102 females (26.8%) reported the same or better PSQ, whereas 347 males (80.9%) and 82 females (19.1%) reported worse PSQ (Table 2). Notably, compared with females, a greater proportion of males experienced worse PSQ during in-hospital PSG.

Among participants reporting ‘same or better’ PSQ, their mean ESS score was 10.0 ± 4.3 compared with 9.3 ± 4.5 among those reporting ‘worse’ PSQ. Moreover, the lower sleepiness scores were significantly associated with worse PSQ compared with higher scores. Apart from gender differences and ESS, no other physiological parameters or comorbidities, including cerebrovascular accident (CVA), were significantly associated with PSQ.

Regarding the effect on perceived sleep quality (PSQ), we found that gender and ESS were independent predictors. Men had an odds ratio (OR) of 1.570 (95% CI: 1.127–2.189) for worse PSQ compared with women (Table 3), while each one-point increase in ESS was associated with an OR of 0.963 (95% CI: 0.933–0.994) for reduction in the risk of worsening PSQ. In other words, participants with greater daytime sleepiness were more likely to report the same or better PSQ during in-hospital PSG.

**Figure 2:**
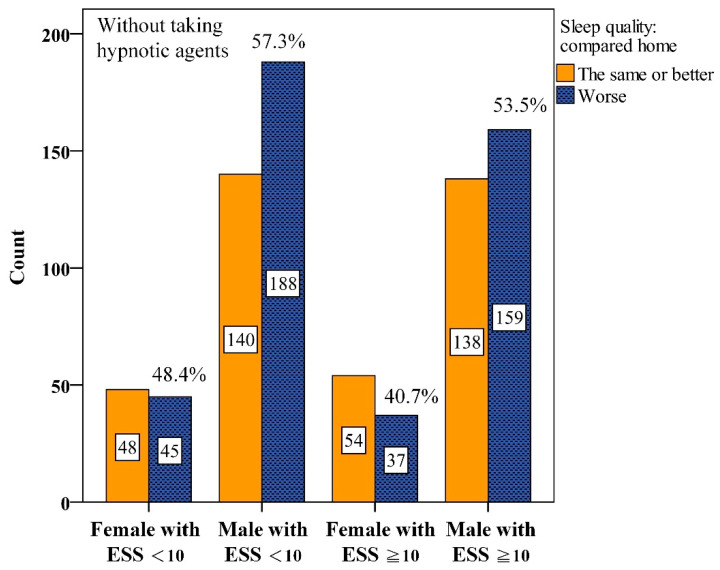
Numbers of interaction among gender and Epworth Sleepiness Scale scores (ESS) for perceived sleep quality without taking hypnotic agents before Polysomnography.

**Figure 3:**
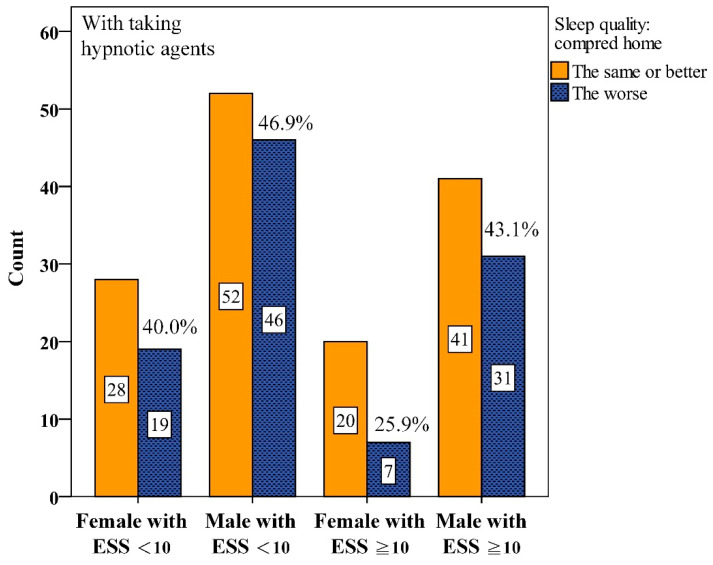
Numbers of interaction among gender and Epworth Sleepiness Scale scores (ESS) for perceived sleep quality with taking hypnotic agents before Polysomnography.

Interactions between gender and ESS were further analyzed. We found that among male participants with ESS < 10, 57.3%, worse PSQ were reported without hypnotics (Figure 2). This proportion dropped to 46.9% with hypnotics (Figure 3). Similarly, among males with ESS ≥ 10, the proportion reporting worse PSQ was 53.5% without hypnotics but dropped to 43.1% with hypnotics.

**Figure 4:**
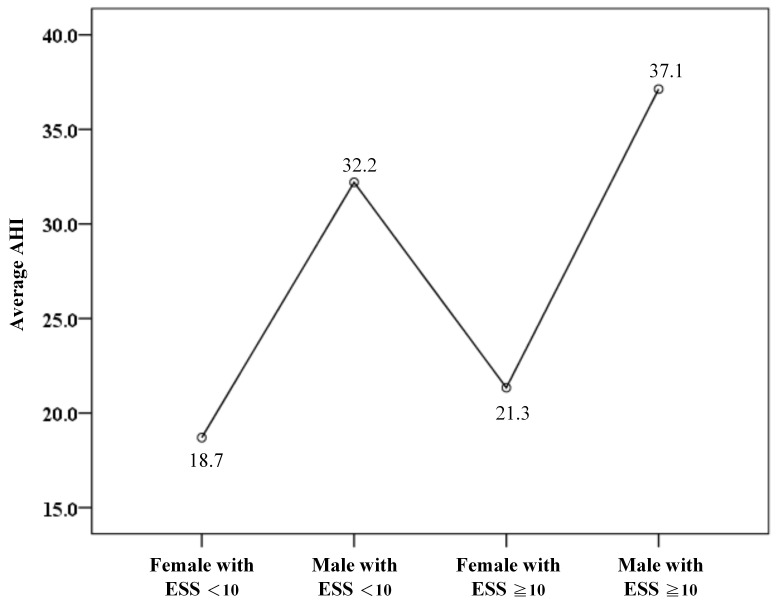
The average apnea–hypopnea index (AHI) among gender and Epworth Sleepiness Scale scores (ESS).

In addition, the mean apnea–hypopnea index (AHI) among men with ESS < 10 was 32.2, which was lower than that of men with ESS ≥ 10 but still remained higher relative to women (Figure 4).

## 4. Discussion

In this large cohort of 1053 adult patients receiving polysomnography (PSG) assessment, several important results emerged from our study. These subjects had an average age of mid-40, with a balanced distribution of body mass index and comorbidities. Male participants were the majority, and approximately 1/4 received hypnotics prior to PSG. These baseline findings highlighted that our study population was representative of a typical sleep-disordered breathing cohort, and the inclusion of both medicated and non-medicated participants allowed assessment on the effect of hypnotics on sleep quality perception in a real-world setting.

According to Table 1, a key observation was that 50.5% of patients reported worse sleep quality in the hospital setting compared with at home. Among those who did not receive hypnotics, 53.0% reported poorer subjective sleep quality, whereas the proportion was 42.2% among patients who received hypnotics. This difference between the two groups was statistically significant. These findings suggest that the hospital sleep environment substantially compromises subjective sleep quality, whereas hypnotics may help mitigate this effect, improving patient comfort and potentially their confidence in the diagnostic validity of PSG. These results align with earlier studies indicating that sedatives can shorten sleep latency and improve perceived sleep continuity during laboratory testing, as supported by Roehrs and Roth (2016), who showed improved sleep onset and reduced nocturnal awakenings with zolpidem [15].The FNE is a well-documented phenomenon that can confound PSG results, leading to underestimation of actual sleep parameters and potentially misrepresenting the severity of underlying sleep disorders [16]. For patients with a high risk of FNE, the discomfort and anxiety associated with the unfamiliar laboratory setting can severely disrupt natural sleep architecture, making it challenging to obtain a representative sleep study. Our data suggest that hypnotics can alleviate this subjective distress, thereby potentially facilitating more stable sleep during the crucial diagnostic night. However, the decision to administer a hypnotic agent for PSG in patients with suspected SDB necessitates a careful consideration of its potential impact on objective diagnostic parameters. While our results indicate benefits in subjective sleep quality and patient acceptance, it is important to recognize that hypnotic agents can alter natural sleep architecture. In particular, certain agents—especially benzodiazepines—are known to affect multiple sleep stages, often reducing slow-wave sleep and potentially altering the proportion of REM sleep [17].

In our investigation, various classes of hypnotic agents—including benzodiazepines, Z-drug, and combined-hypnotic therapy—were evaluated for their impact on perceived sleep quality before PSG. While benzodiazepines typically reduce slow-wave and REM sleep while increasing NREM stage 2 sleep, and Z-drug tend to better preserve natural sleep architecture, no specific class demonstrated a statistically superior effect on subjective sleep quality (*p* = 0.569). This suggests that the primary benefit of these agents lies in their shared sedative or anxiolytic properties, contributing to comparable improvements in sleep quality across different classes. Therefore, the judicious use of hypnotics can enhance patient comfort and trust in in-laboratory sleep studies, with drug selection guided by safety profile, rapid onset, and individual tolerance, rather than an expectation of class-specific superior efficacy [18,19,20].

A particular concern in the diagnosis of SDB is the possibility that hypnotic agents may exacerbate upper airway collapsibility. By suppressing central nervous system activity and muscle tone, these agents may lead to an overestimation of the AHI and/or worsen desaturation status during the PSG study. A falsely elevated AHI could lead to an inappropriate or overly aggressive treatment plan. Conversely, in cases of severe FNE-induced sleep fragmentation, a hypnotic might facilitate more consolidated sleep, paradoxically “unmasking” SDB events that might otherwise be missed if the patient is mostly awake or in very light sleep.

Therefore, the recommendation for using a hypnotic agent in SDB patients at high risk for FNE should not be a routine practice but rather a deliberate, individualized decision made under the premise of maximizing patient benefit while acknowledging potential diagnostic pitfalls. Clinicians must carefully weigh the psychological benefit of improved comfort and trust in the diagnostic process against the physiological risk of altering SDB severity metrics. If the use of a hypnotic is deemed necessary, short-acting agents with minimal known effects on respiratory drive and sleep architecture should be preferred. Full disclosure to the patient regarding the potential influence of the medication on PSG results is paramount. Furthermore, the interpretation of PSG data obtained under the influence of hypnotics must be approached with caution, recognizing the potential for biased AHI and desaturation values.

These findings carry meaningful clinical implications for optimizing diagnostic strategies in sleep medicine. When the primary aim is to screen for obstructive sleep apnea (OSA), central sleep apnea (CSA), or mixed sleep apnea, home sleep apnea testing (HSAT) may be considered a practical and patient-friendly alternative. Many individuals tend to experience better sleep quality and reduced anxiety in their home environment compared with the hospital setting, which may enhance test validity and increase their confidence in the diagnostic results. Such improved trust and comfort could, in turn, facilitate earlier treatment acceptance and adherence [21]. Nevertheless, this approach should be applied with caution in specific patient populations. In individuals with co-morbid insomnia and sleep apnea (COMISA), difficulty initiating or maintaining sleep during home testing may increase the likelihood of false-negative results. For these patients, in-lab polysomnography remains the preferred and more reliable diagnostic modality [22].

Taken together, while HSAT represents a valuable option for many patients with a primary suspicion of OSA—especially those who sleep more naturally at home—it should be complemented by careful clinical judgment to identify subgroups, such as COMISA, who may benefit more from in-lab assessment. From a system-level perspective, the extensive use of HSAT in primary care or family medicine settings could further help ease the typically long waiting time for patients typically at the hospital-based sleep laboratory, thereby improving access and efficiency of care [23].

We also found that patients receiving sleeping pills paradoxically exhibited shorter SDT, longer SL, and lower SE compared with those without such medications. This response pattern likely reflected underlying patient characteristics rather than a direct pharmacologic effect of hypnotics. Specifically, individuals with greater baseline anxiety or insomnia symptoms were likely more inclined to request or accept hypnotics before PSG. Similar findings were reported earlier in both psychiatric and geriatric populations, where patients with higher anxiety are more likely to receive sleeping medications, independent of objective sleep parameters [24]. Anxiety is well known to prolong sleep latency and reduce sleep efficiency, and such symptoms often drive patients toward pharmacologic sleep aids [25]. Therefore, our results suggest that the poorer objective sleep indices observed in the medicated group likely reflected the pre-existing vulnerability of anxious patients rather than medication effects. Clinically, this finding highlighted the importance of carefully evaluating psychological traits during pre-PSG assessment. For patients with prominent anxiety features—whether identified through clinical interview or standardized questionnaires—a short-acting hypnotic agent may improve their subjective tolerance of the PSG study and acceptance of PSG results and the reliability of their test outcomes.

Gender-related differences were also evident. Male patients showed a 1.570-fold higher likelihood of reporting worse sleep compared with females. This finding is consistent with contemporary and legacy reports showing that, the in the obstructive sleep apnea (OSA) cohorts, men exhibit shorter total sleep time, lower efficiency, and more frequent arousals on PSG than women. By contrast, women often display longer sleep latency but a relatively higher proportion of slow-wave sleep, with patterns seen both in patient samples and in healthy young sleepers. At the same time, survey-based psychometric studies indicated that women report poorer global sleep quality on questionnaires despite more favorable PSG indices, underscoring a subjective–objective dissociation that is partially gender-dependent. Our findings add clarity to this picture by showing that in a hospital-based PSG setting, male gender is a stronger predictor of poor subjective sleep quality [26,27].

The interaction with the Epworth Sleepiness Scale (ESS) further refines the above picture. Individuals with lower ESS scores (<10) reflecting minimal daytime sleepiness were more likely to report worse subjective sleep quality during PSG, and each one-point increase in ESS was associated with a 0.963 reduction in the odds of reporting worse sleep. This seemingly paradoxical pattern is consistent with evidence that ESS (daytime sleepiness) and PSQI (nighttime sleep quality) capture related but distinct constructs which are weakly correlated. Patients who are less habitually sleepy are likely more prone to environmental disturbance, whereas those with greater baseline sleepiness may be less affected during PSG [28].

Beyond gender and ESS, we found no significant associations between perceived sleep quality and age, BMI, or cardiovascular comorbidities. This finding emphasizes that subjective perception of sleep during PSG is shaped not much by traditional risk factors but more by demographic and psychological factors. Compared with earlier literature, our study has several strengths: the large sample size improves statistical precision; the joint analysis of questionnaire-based perceptions with PSG parameters offers a more holistic view of the patient experience; and the explicit examination of interactions among hypnotic use, gender, and ESS identifies those subgroups most vulnerable to poor subjective experience—yielding clinically practical insights that extend beyond prior descriptive work on gender differences in PSG architecture and symptom presentation [26].

The post-PSG questionnaire was routinely used in our hospital’s clinical practice to assist in interpreting PSG results. The three independent components were the time to fall asleep, perceived sleep quality, and perceived sleep duration. A previous study reported a first-night effect on PSG in the hospital setting of 48.9%, which closely matched our statistical data and aligns with established academic post hoc content validity standards [29].

The clinical implications are clear. Patients who believe they slept poorly during PSG may doubt the validity of the test and are hesitant to accept treatment recommendations. Our results suggest those male patients and those with ESS < 10 are at highest risk for such perceptions. For these individuals, clinicians should provide anticipatory counseling regarding the first-night effect and reassure them the reliability of PSG as a diagnostic tool despite the poorly perceived sleep. Selective, short-term use of hypnotics is reasonable to improve subjective sleep quality and acceptance of results, particularly in these vulnerable subgroups that provided use is judicious while avoiding routine long-term administration. Evidence has indicated acute benefits and no gender-based differences in hypnotic efficacy with chronic nightly use under well-monitored conditions, whereas long-term, non-targeted hypnotic use is associated with tolerance, dependence, and degraded sleep quality [15,16,17].

In addition, it was a retrospective, single-center analysis conducted at Taichung Veterans General Hospital, and the assessment of sleep quality relied on post-PSG questionnaires, which are inherently subjective and prone to recall bias. Although adjustments were made for common confounders such as age, BMI, and comorbidities, we were unable to incorporate objective physiological markers such as arousal thresholds or perform subgroup analyses for patients with low arousal threshold phenotypes.

In addition, the post-PSG questionnaire did not account for environmental and behavioral factors that may substantially influence sleep quality, including ambient light conditions, light exposure timing, and circadian light hygiene. Given that hospital illumination may differ considerably from home environments, the first-night effect could be exaggerated by such lighting discrepancies. Moreover, physical activity levels—such as the amount, duration, timing, and regularity of daily movement prior to PSG—were not recorded. Reduced activity on testing days might have altered sleep latency or efficiency, potentially mimicking or amplifying the first-night effect.

While adjustments were made for demographic variables, age-related effects were not specifically analyzed. To confirm age-stratified effects, statistical comparisons between individuals aged ≥65 and those under 65 were conducted. However, there was no significant difference in perceived sleep quality between these groups, despite the possibility that older adults may experience more pronounced first-night effects due to comorbidities or circadian rhythm alterations. Additionally, other contextual factors such as bed size at home, pillow comfort, mattress characteristics, and room temperature were not assessed; these factors could influence nocturnal posture and body movements, further contributing to differences in perceived sleep quality.

Finally, we did not capture the specific type or dosage of hypnotic medications used by participants, nor did our cohort include other commonly used classes such as dual orexin receptor antagonists (DORAs), melatonin-based agents, sedative antidepressants (except mirtazapine), or sedative antipsychotics. As a result, our findings cannot be generalized to all hypnotic classes, which represent an important limitation in interpreting drug-related effects on both objective and subjective sleep quality. The generalizability of the findings is also restricted to hospital-based cohorts. Future multicenter, prospective studies with multimodal assessment—including environmental monitoring, activity tracking, and objective sleep parameters—are warranted to validate these findings, clarify the influence of age, environmental, and pharmacologic factors, and further define the role of short-term hypnotics as targeted interventions, particularly among men with low ESS [25,30].

## 5. Conclusions

Results of our study highlighted two important clinical implications in optimizing the diagnostic process of sleep-disordered breathing. First, for patients without a history of stroke or significant cardiovascular risk, HSAT may be considered as the initial diagnosed tool for sleep apnea. Conducting the study in the home environment may improve sleep quality and patient confidence in the diagnostic reports. When HSAT fails to explain excessive daytime sleepiness or when alternative sleep disorders are suspected, a subsequent in-laboratory polysomnography remains essential for differential diagnosis.

Second, our findings indicated that male patients with low Epworth Sleepiness Scale scores (ESS < 10) and anxiety traits are more likely to report poor subjective sleep quality during in-laboratory PSG. For this subgroup, the judicious use of short-acting hypnotic agents may be considered to reduce the likelihood of poor subjective sleep perception, thereby improving the acceptability and reliability of PSG results. Together, these strategies can enhance patient-centered care by tailoring diagnostic approaches to both physiological and psychological patient characteristics.

## Figures and Tables

**Table 1:** Baseline demographics and clinical characteristics of enrolled participants.

	All (*n* = 1053)	With Hypnotic Agents ^κ^ (*n* = 244)	Without Hypnotic Agents (*n* = 809)	*p*-Value	Effect Size/Power
Gender					
Male	795 (75.5)	170 (69.7)	625 (77.3)	0.020 *	0.074/0.67
Female	258 (24.5)	74 (30.3)	184 (22.7)		
Age	47.0 ± 14.7	52.8 ± 13.8	46.2 ± 14.6	0.000 *	0.45/0.99
BMI (kg/m^2^)	26.5 ± 4.6	25.9 ± 4.5	26.7 ± 4.6	0.023 *	
<25.0	431 (40.9)	111 (45.5)	320 (39.6)	0.033 *	0.081/0.75
≧25.0, <30.0	426 (40.5	101 (41.4)	325 (40.2)		
≧30.0	196 (18.6)	32 (13.1)	164 (20.3)		
ESS	9.5 ± 4.4	8.8 ± 4.4	9.7 ± 4.4	0.012 *	0.20/0.86
≧10.0	487 (46.2)	99 (40.6)	388 (48.0)	0.051	
<10.0	566 (53.8)	145 (59.4)	421 (52.0)		
HBP					
Yes	285 (27.1)	69 (28.3)	216 (26.7)	0.686	
No	768 (72.9)	175 (71.7)	593 (73.3)		
CVA					
Yes	28 (2.7)	6 (2.5)	22 (2.7)	1.000	
No	1025 (97.3)	238 (97.5)	787 (97.3)		
AHI	29.7 ± 26.1	24.7 ± 21.8	31.2 ± 27.1	0.000 *	0.25/0.96
SDT (min)	342.4 ± 33.5	337.0 ± 39.3	344.0 ± 31.4	0.012 *	0.21/0.89
TST (min)	303.5 ± 51.3	298.9 ± 48.5	305.0 ± 52.1	0.104	
SL (min)	18.9 ± 22.8	24.6 ± 27.0	17.1 ± 21.1	0.000 *	0.33/0.99
SE (%)	83.5 ± 13.8	81.7 ± 13.2	84.0 ± 13.9	0.024 *	0.17/0.75
Stage N1 (%)	19.2 ± 15.2	20.7 ± 16.8	18.7 ± 14.6	0.062	
Stage N2 (%)	62.4 ± 22.0	63.9 ± 15.1	61.9 ± 23.7	0.212	
Stage N3 (%)	7.0 ± 8.8	5.3 ± 8.8	7.4 ± 8.7	0.001 *	0.24/0.95
Stage R (%)	11.9 ± 6.5	9.8 ± 5.6	12.6 ± 6.6	0.000 *	0.43/0.99
LTFA					
The same or shorter	692 (65.7)	168 (68.9)	524 (64.8)	0.271	
The longer	361(34.3)	76 (31.3)	285 (35.2)		
PSDT					
The shorter	538 (51.1)	112 (45.9)	426 (52.7)	0.076	
The same or longer	515 (48.9)	132 (54.1)	383 (47.3)		
PSQ					
The same or better	521 (49.5)	141 (57.8)	380 (47.0)	0.004 *	0.091/0.84
The worse	532 (50.5)	103 (42.2)	429 (53.0)		

LTFA: Length of time to fall asleep; PSDT: Perceived sleep duration time; PSQ: perceived sleep quality. BMI: body mass index; ESS: Epworth sleepiness scale; HBP: high blood pressure. CVA: Cerebrovascular accident; AHI: apnea–hypopnea index; SDT: sleep duration time; TST: total sleep time; SL: sleep latency; SE: sleep efficiency. Effect size presented with Cramer’s V for category variables, Cohen’s d for continuous variables (α = 0.05). κ: The investigation for the types of hypnotic agents using (see Table S1 and Figure S1 of the supplementary). * *p* < 0.05.

**Table 2:** Parameter comparisons between same or better and worse for perceived sleep quality.

	Same or Better (*n* = 380)	Worse (*n* = 429)	*p*-Value	Effect Size/Power
Gender				
Male	278 (73.2)	347 (80.9)	0.011 *	0.092/0.74
Female	102 (26.8)	82 (19.1)		
Age	46.4 ± 15.2	46.0 ± 14.1	0.715	
≧65	52 (13.7)	49 (11.4)	0.387	
<65	328 (86.3)	380 (88.6)		
BMI (Kg/m^2^)	26.9 ± 4.6	26.5 ± 4.7	0.269	
<25.0	137 (36.1)	183 (42.7)	0.153	
≧25.0, <30.0	163 (42.9)	162 (37.8)		
≧30.0	80 (21.1)	84 (19.6)		
ESS	10.0 ± 4.3	9.3 ± 4.5	0.025 *	0.16/0.73
HBP				
Yes	112 (29.5)	104 (24.2)	0.110	
No	268 (70.5)	325 (75.8)		
CVA				
Yes	10 (2.6)	12 (2.8)	1.000	
No	370 (97.4)	417 (97.2)		

BMI: body mass index; ESS: Epworth sleepiness scale; HBP: high blood pressure. CVA: Cerebrovascular accident. Effect size presented with Cramer’s V for category variables, Cohen’s d for continuous variables (α = 0.05). * *p* < 0.05.

**Table 3:** Regression model of the significant factors and potential confounders for perceived sleep quality.

**Simple Logic Regression**		
**Factors**	**Odds Ratio (95% CI)**	***p*** **-Value**
Male vs. female	1.553 (1.115–2.161)	0.009 *
Per increasing 1 ESS score	0.964 (0.934–0.995)	0.025 *
Age increasing 1 year-old	0.998 (0.989–1.008)	0.713
BMI increasing 1 kg/m^2^	0.983 (0.954–1.013)	0.269
AHI increasing 1 index	1.002 (0.997–1.007)	0.396
**Multiple Logic Regression**		
**Factors**	**Odds Ratio (95% CI)**	***p*** **-Value**
Male vs. female	1.570 (1.127–2.189)	0.008 *
Per increasing 1 ESS score	0.963 (0.933–0.994)	0.021 *

ESS: Epworth sleepiness scale; BMI: body mass index; AHI: apnea–hypopnea index. * *p* < 0.05

## Data Availability

The raw data supporting the conclusions of this article will be made available by the authors on request.

## Supplementary Materials

The following supporting information can be downloaded at: https://www.mdpi.com/article/10.3390/diagnostics15243153/s1, Figure S1: Investigation results of the types of hypnotic agents used before Polysomnography diagnosis based on the post-questionnaires (available numbers were 153); Table S1: Differences in perceived sleep quality across hypnotic agents.

## Abbreviations

The following abbreviations are used in this manuscript:

LTFA	Length of time to fall asleep
PSDT	Perceived sleep duration time
PSQ	Perceived sleep quality
BMI	Body mass index
ESS	Epworth sleepiness scale
HBP	High blood pressure
CVA	Cerebrovascular accident
AHI	Apnea–hypopnea index
SDT	Sleep duration time
TST	Total sleep time
SL	Sleep latency
SE	Sleep efficiency
SQ	Sleep quality
