# Supplementary material for: Discrepancies in Subjective Sleep Quality Between Home and Hospital Settings: Insights of Hypnotic Agents Use with Post-Polysomnography Questionnaire
Source: Diagnostics (Basel). 2025 Dec 11;15(24):3153. doi: 10.3390/diagnostics15243153 (PMC12732203; doi:10.3390/diagnostics15243153)
Supplement: Supplementary file 1 [file diagnostics-15-03153-s001.zip › diagnostics-3935959-supplementary.pdf]

Table S1. Differences in perceived sleep quality across hypnotic agents.

	**Same or better (N=93)**	**Worse (N=60)**	***p*- value**
Benzodiazepines	17 (18.3)	15 (25.0)	0.569
Z drugs	43 (46.2)	27 (45.0)	
Combined Hypnotics	33 (35.5)	18 (30.0)	

Benzodiazepines included agents such as alprazolam or estazolam, whereas Z-drugs included agents such as zopiclone or zolpidem. Combined-hypnotic therapy was defined as the concomitant use of two or more agents from benzodiazepines, Z-drugs, or sedative antidepressants. The detailed distribution of hypnotic agents was presented in Figure S1. P-values were calculated using the Chi-Square test.

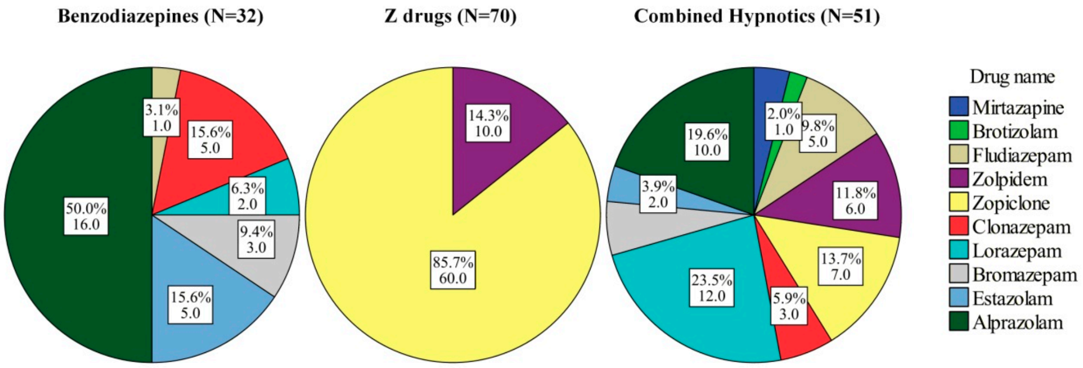

Figure S1. The investigation for the types of hypnotic agents using before Polysomnography diagnosis based on the post-questionnaires (available numbers were 153).
